# Insights into adolescents' brain health perception: A research study

**DOI:** 10.1002/alz70860_101071

**Published:** 2025-12-23

**Authors:** Temitope Hannah Farombi, Olajoke Sarah Akinyemi, Joaquín Migeot, Olubukola Ebunoluwa Ojelade, Olufisayo Oluyinka Elugbadebo

**Affiliations:** ^1^ University College Hospital, Ibadan, Nigeria; ^2^ Global Brain Health Inistitute, Trinity College Dublin, Dublin, Dublin, Ireland; ^3^ Brain Health Initiative Nigeria, Ibadan, Oyo State, Nigeria; ^4^ Latin American Brain Health Institute (BrainLat), Universidad Adolfo Ibañez, Santiago, Chile; ^5^ Center for Social and Cognitive Neuroscience, Universidad Adolfo Ibáñez, Santiago, Chile; ^6^ Latin American Brain Health Institute (BrainLat), Universidad Adolfo Ibáñez, Santiago, Región Metropolitana de Santiago, Chile; ^7^ College of Medicine, University of Ibadan, Ibadan, Nigeria

## Abstract

**Background:**

Adolescence is often thought to be a period of heightened risk‐taking in activities that are detrimental to brain health. These include unhealthy eating, substance use, fighting, truancy, and vandalism. Despite the vulnerability, little is known about the knowledge and perception of adolescents on the risk factors for brain health. This study is part of a larger research aimed at educating adolescents on the knowledge of brain health. The objective of this study is to investigate the perception of adolescents on brain health.

**Method:**

This pilot study recruited 151 students in southwestern Nigeria from both public and private secondary schools to allow engagement from diverse settings. At the baseline, randomly sampled students participated in a perception survey. Data was analyzed with SPSS (v. 26).

**Result:**

Analysis revealed that 59.2% were female, 54.6% were Christians, and 88.8% were Yoruba. Students’ mean age was 13.4±0.5. While 60.3% believed that alcohol and smoking could harm the brain, 41.7% of the respondents did not view social isolation as a risk factor for brain health. Surprisingly, 41.1% disagreed that a healthy diet could improve thinking abilities.

**Conclusion:**

Our study provides insights into important gaps in brain health perceptions among Nigerian adolescents. Consequently, there is a need to address these gaps by providing awareness through collaborative efforts of Governmental and Non‐Governmental institutions.

